# A Ten-microRNA Expression Signature Predicts Survival in Glioblastoma

**DOI:** 10.1371/journal.pone.0017438

**Published:** 2011-03-31

**Authors:** Sujaya Srinivasan, Irene Rosita Pia Patric, Kumaravel Somasundaram

**Affiliations:** Department of Microbiology and Cell Biology, Indian Institute of Science, Bangalore, Karnataka, India; University of Birmingham, United Kingdom

## Abstract

Glioblastoma (GBM) is the most common and aggressive primary brain tumor with very poor patient median survival. To identify a microRNA (miRNA) expression signature that can predict GBM patient survival, we analyzed the miRNA expression data of GBM patients (n = 222) derived from The Cancer Genome Atlas (TCGA) dataset. We divided the patients randomly into training and testing sets with equal number in each group. We identified 10 significant miRNAs using Cox regression analysis on the training set and formulated a risk score based on the expression signature of these miRNAs that segregated the patients into high and low risk groups with significantly different survival times (hazard ratio [HR] = 2.4; 95% CI = 1.4–3.8; p<0.0001). Of these 10 miRNAs, 7 were found to be risky miRNAs and 3 were found to be protective. This signature was independently validated in the testing set (HR = 1.7; 95% CI = 1.1–2.8; p = 0.002). GBM patients with high risk scores had overall poor survival compared to the patients with low risk scores. Overall survival among the entire patient set was 35.0% at 2 years, 21.5% at 3 years, 18.5% at 4 years and 11.8% at 5 years in the low risk group, versus 11.0%, 5.5%, 0.0 and 0.0% respectively in the high risk group (HR = 2.0; 95% CI = 1.4–2.8; p<0.0001). Cox multivariate analysis with patient age as a covariate on the entire patient set identified risk score based on the 10 miRNA expression signature to be an independent predictor of patient survival (HR = 1.120; 95% CI = 1.04–1.20; p = 0.003). Thus we have identified a miRNA expression signature that can predict GBM patient survival. These findings may have implications in the understanding of gliomagenesis, development of targeted therapy and selection of high risk cancer patients for adjuvant therapy.

## Introduction

The grade IV astrocytoma, GBM, is the most common and malignant primary adult brain cancer [1]. Despite advances in treatment modalities, the median survival is very poor. Since postoperative radiotherapy alone did not provide great benefit to GBM patients, several attempts have been made to find suitable adjuvant chemotherapy. The present standard treatment appears to be maximal safe resection of the tumor followed by irradiation and temozolomide adjuvant chemotherapy [2]. However, it was found that not all patients were benefited from the addition of temozolomide. Further analysis revealed that methylation of MGMT promoter to be the strongest predictor for outcome and benefit from temozolomide chemotherapy [2]. In addition, recent molecular and genetic profiling studies have identified several markers and unique signatures as prognostic and predictive factors of GBM [3], [4].

MicroRNAs (miRNAs) are endogenous non-coding small RNAs, which negatively regulate gene expression either by binding to the 3′ UTR leading to inhibition of translation or degradation of specific mRNA. Since miRNAs can act as Oncogenes or tumor suppressor genes, they have been linked to a variety of cancers [5]. It has been shown that classification of multiple cancers based on miRNA expression signatures is more accurate than mRNA based signatures [6]. There have been a few attempts to profile miRNA expression either by microarray or RT-PCR in different grades of glioma [7]–[11]. Rao et al., profiled the expression of 756 miRNAs using 39 malignant astrocytoma and 7 normal brain samples and identified a 23-miRNA expression signatures which can discriminate anaplastic astrocytoma from glioblastoma [11]. Other studies investigated the target identification and functional characterization of specific miRNAs [8], [10], [12]–[17]. Many studies identifying miRNA expression signatures predicting patient survival have been done in several cancers like lung cancer, lymphocytic leukemia; lung adenocarcinoma, breast and pancreas cancers [18]–[23]. However, a miRNA signature that can predict the clinical outcome in GBM patients has not been found so far.

In this study, we have subjected the miRNA expression data from a total of 222 GBM patients derived from The Cancer Genome Atlas (TCGA) data set to Cox proportional regression analysis to identify the miRNAs that can predict patient survival. By using a sample-splitting approach, a 10 miRNA expression signature that can predict survival both in training and testing sets was identified. More importantly, using multivariate analysis along with patient age, the 10 miRNA expression signature was found to be an independent predictor of patient survival.

## Results

### Identification of a 10 miRNA expression signature from training set

**Figure 1:**
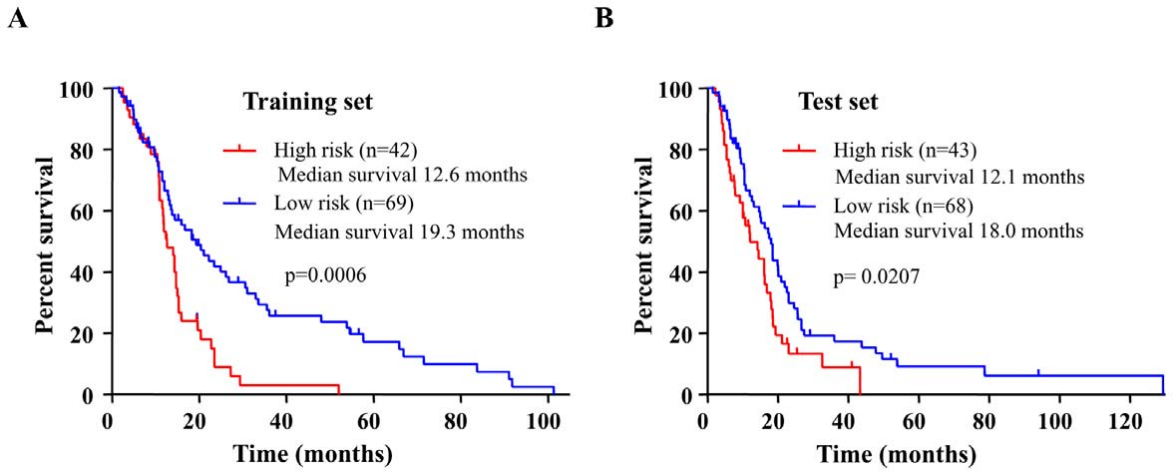
Kaplan-Meier survival estimates overall survival of glioblastoma patients according to the 10 miRNA expression signature. **A**) 111 GBM patients in the training data set. **B**) 111 GBM patients in the testing data set.

The 222 GBM samples were divided randomly into a training set (n = 111) or a testing set (n = 111). Table 1 gives the age and gender distribution of the patients in both sets and the entire set. miRNA expression data corresponding to 305 miRNAs derived from the training set was subjected to Cox proportional hazard regression analysis to identify miRNAs, whose expression profile could be significantly correlated to patient survival. We identified a set of 10 miRNAs that were significantly correlated with patient survival (**Table 2**). These 10 miRNAs were then used to create a signature by calculating a risk score for each patient. A risk score formula was obtained for predicting the patient survival (see materials and methods for detail). Using the risk score formula, the 10 miRNA expression signature risk score was calculated for all patients in the training set. The patients were then ranked in the training set according to their risk score. Using the 60^th^ percentile risk score as cutoff in the training set, the patients were divided into high and low risk groups. Patients belonging to high risk group had a shorter median survival than patients with low risk score (12.6 months versus 19.3 months, HR = 2.4; 95% CI = 1.4–3.8; p = 0.0006) (**Figure 1** **A;** **Table 3**). Survival was greater in the low risk group than in the high risk group throughout the follow-up. Overall survival in the training set was 41.8% at 2 years, 25.6% at 3 years, 23.6% at 4 years and 14.8% at 5 years in the low risk group, versus 9.0%, 3.0%, 0.0 and 0.0% respectively in the high risk group (HR = 2.4; 95% CI = 1.4–3.8; p = 0.0006) (**Table 3** **; Figure S1**).

**Table 1:** Clinical characteristics of GBM patients according to their low or high risk group in the training set, the testing set and the entire patient set.

Characteristic	Training set		*p-value* *	Testing set		*p-value* *	Entire patient set		*p-value* *
	Low risk (N = 69)	High risk (N = 42)		Low risk (N = 68)	High risk (N = 43)		Low risk (N = 137)	High risk (N = 85)	
Age (Mean ± SD)	51.5±6.6	54.4±1.9	0.5	54±12.7	58.3±10.2	0.1	52.8±14.8	56.4±11.2	0.1
Gender									
Female (%)	26 (37.7)	13 (31)	0.5	27 (39.7)	12 (27.9)	0.2	53 (38.7)	25 (29.4)	0.2
Male (%)	43 (62.3)	29 (69)		41 (60.3)	31 (72.1)		84 (61.3)	60 (70.6)	

*Two-tailed *p-value* obtained from Mann-Whitney test.

**Table 2:** Ten miRNA signature that predicts survival in glioblastoma patients.

No	miRNA	Type	Hazard Ratio	Coefficient	Permutation *p-value*	Median log2 ratio#		GBM Vs Normal	
						Low risk	High risk	Regulation$	*p-value* *
1	hsa-miR-20a	**Protective**	0.68	−0.39	0.0004	1.83	0.86	upregulated	<0.0001
2	hsa-miR-106a		0.66	−0.41	0.0005	1.67	0.65	upregulated	<0.0001
3	hsa-miR-17-5p		0.68	−0.39	0.0008	1.60	0.69	upregulated	<0.0001
4	hsa-miR-31	**Risky**	1.32	0.28	0.0001	−1.83	−0.82	downregulated	0.0022
5	hsa-miR-222		1.26	0.23	0.0004	−2.15	−0.70	downregulated	0.0002
6	hsa-miR-148a		1.21	0.19	0.0010	1.16	2.83	upregulated	<0.0001
7	hsa-miR-221		1.27	0.24	0.0014	−1.93	−0.53	downregulated	0.0003
8	hsa-miR-146b		1.25	0.22	0.0025	0.10	1.22	upregulated	<0.0001
9	hsa-miR-200b		1.21	0.19	0.0029	−0.02	0.83	upregulated	<0.0001
10	hsa-miR-193a		1.34	0.29	0.0045	1.05	2.10	upregulated	<0.0001

# Median of the log 2 transformed ratios of GBM vs Normal.

$ type of regulation (upregulated or downregulated) in GBM in comparison to Normal.

*Two-tailed *p-value* obtained from Mann-Whitney test.

**Table 3:** Kaplan Meier overall survival analysis in the training set, the testing set and the entire patient set.

Patient set	Deaths/Patient (%)	Hazard Ratio (95% CI)	Median survival (months, 95% CI)	Survival (95% CI)				
				1 year (%)	2 years (%)	3 years (%)	4 years (%)	5 years (%)
**Training set**								
Low risk (n = 69)	57/69 (82.6)	1	19.3 (12.7–25.9)	66.5 (54.9–78.1)	41.8 (29.5–54.1)	25.6 (14.5–6.7)	23.6 (12.6–34.6)	14.8 (5–24.6)
High risk (n = 42)	38/42 (90.5)	2.4 (1.4–3.8)	12.6 (10.1–15.1)	50.6 (35.1–6.1)	9.0 (0.0–18.4)	3.0 (0.0–8.7)	0.0	0.0
**Test set**								
Low risk (n = 68)	55/68 (80.9)	1	18.0 (14.5–21.5)	64.8 (52.8–76.8)	28 (16.4–39.6)	17.3 (7.5–27.1)	13.5 (5.1–21.9)	6.2 (0–13.5)
High risk (n = 43)	36/43 (83.7)	1.7 (1.1–2.8)	12.1 (6.8–17.4)	49.8 (34.5–64.1)	13.3 (2.2–24.4)	8.9 (0.0–19.3)	0.0	0.0
**Entire patient set**								
Low risk (n = 137)	112/137 (81.8)	1	18.3 (15–21.6)	65.6 (57.4–72.8)	35.0 (26.4–3.2)	21.5 (14.1–28.9)	18.5 (11.2–25.8)	11.8 (5.5–18.1)
High risk (n = 85)	74/85 (87.1)	2.0 (1.4–2.8)	12.6 (10.3–14.9)	51.4 (40.6–62.2)	11.0 (3.5–18.5)	5.5 (0–11.2)	0.0	0.0

### Validation of the 10 miRNA expression signature for survival prediction in the testing set

To validate our finding, we calculated the risk score for the 111 patients from the testing set. Using the same cut-off value that was used for training set, the patients from the testing set were classified into low risk and high risk groups and subjected to survival comparison. Similar to the results obtained in the training set, patients in the high risk group had shorter median survival than patients in the low risk group (12.1 months versus 18.0 months; HR = 1.7; 95% CI = 1.1–2.8; p = 0.0207) (**Figure 1** **B;** **Table 3**). As was seen in the training set, patient survival in the low risk group was better than that in the high-risk group throughout the 5 year follow-up time (**Table 3**). Risk score based classification of the entire patient set also gave a similar result with the high risk group having a shorter median survival than the low risk group (12.6 months versus 18.3 months, HR = 2.0; 95% CI = 1.4–2.8; p<0.0001) (**Table 3**). The higher overall survival of the low risk group throughout the study period in training, testing and entire patient sets compared to the high risk group is shown (**Figure S1**).

### Nature of 10 miRNA expression signature

**Figure 2:**
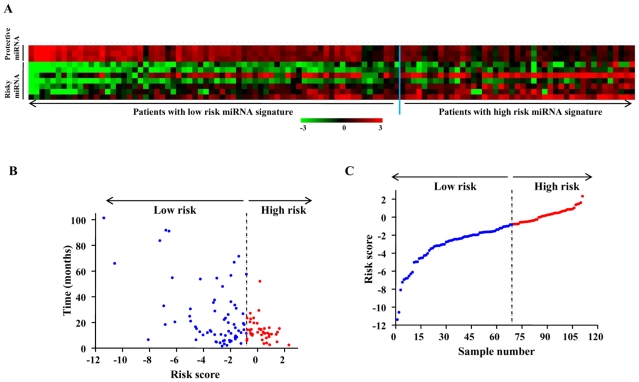
Ten miRNA Risk-Score Analysis of 111 GBM patients (training set). **A**) Heat map of ten miRNA expression profiles of GBM patients; rows represent risky and protective miRNAs, and columns represent patients. The blue line represents the miRNA signature cutoff dividing patients into low-risk and high-risk groups. **B**) Patient survival status along with risk score. **C**) miRNA risk-score distribution of the GBM patients.

**Figure 3:**
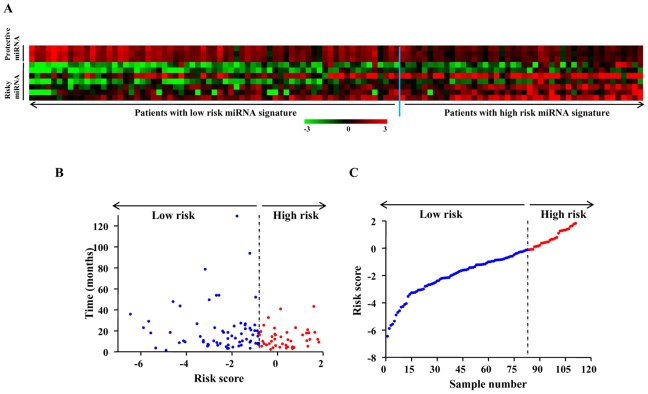
Ten miRNA Risk-Score Analysis of 111 GBM patients (test set). **A**) Heat map of ten miRNA expression profiles of GBM patients; rows represent risky and protective miRNAs, and columns represent patients. The blue line represents the miRNA signature cutoff dividing patients into low-risk and high-risk groups. **B**) Patient survival status along with risk score. **C**) miRNA risk-score distribution of the GBM patients.

Additional investigation of 10 miRNA set yielded several interesting observations. The distribution of miRNA expression values, patient risk scores and survival status of patients were analyzed independently for both training and testing set (**Figure 2** **and** **3**). There were 3 miRNAs that were protective and 7 miRNAs that were risky based on correlation of their expression and association with patient survival (**Table 2**). Tumors from patients belonging to high risk group tend to express higher levels of risky miRNAs, whereas tumors from patients with low risk group tend to express higher levels of protective miRNAs (**Figures 2A**). Similar results were obtained in the testing set as well (**Figure 3A**). A comparison of risk score with patient survival status and risk score distribution among GBM patients of the training set and test set are shown (**Figure 2** **B, C and 3 B, C**). It is interesting to note that the three protective miRNAs are up regulated in glioblastomas (GBMs) compared to normal samples (**Table 2**). However among risky miRNAs, three were down regulated and four were up regulated in GBMs (**Table 2**).

We further carefully analyzed the 10 miRNA set to determine whether a subset of miRNAs can be used to predict GBM patient survival. The four most significant miRNAs from the set of 10, all with permutation p≤0.0005 with (miR-20a, miR-106A, miR-31 and miR-222) were chosen and their expression signature derived risk score was used to predict GBM patient survival. The results showed that unlike the ten miRNA signature, the four miRNA did not consistently correlate with patient survival in training and testing set (**data not shown**).

### Multivariate regression analysis shows that the 10 miRNA expression signature is independent of age

In order to ascertain whether the 10 miRNA expression signature based risk score is an independent predictor of GBM patient's survival, we carried out Cox multivariate analysis. As has been shown before, patient age also predicted the GBM patients survival in univariate analysis (p<0.0001; HR = 1.027; B = 0.027) (**Table 4**). The effect of risk score and age on GBM patient survival was further evaluated by multivariate Cox proportional hazard model. We found that both risk score (p = 0.003; HR = 1.120; B = 0.113) and age (p = 0.004; HR = 1.020; B = 0.019) are independent predictors of GBM patient survival (**Table 4**).

**Table 4:** Cox regression analysis of risk score and age in the entire patient set (n = 222).

Variable	Hazard ratio (95% CI)	*p-value* *
**Univariate analysis**		
Risk score	1.17 (1.09–1.25)	<0.0001
Age	1.03 (1.02–1.04)	<0.0001
**Multivariate analysis**		
Risk score	1.12 (1.04–1.20)	0.003
Age	1.02 (1.01–1.03)	0.004

*Two-tailed *p-value* obtained from Mann-Whitney test.

## Discussion

In this study, we have identified a ten miRNA signature that is associated with survival of GBM patients. We confirmed these findings in a testing set. Patients with a high risk score had shorter survival even after including patient age as a variable in a multivariate Cox model. These results suggest that miRNAs play an important role in molecular pathogenesis, progression and prognosis of GBMs.

Predicting the benefit of various cancer therapies to patients is very important and forms the foundation of personalized cancer therapy. While the clinical features like age and Karnofsky performance status are known prognostic markers among GBM patients, MGMT gene promoter methylation status is of great interest in recent times because it predicted response of GBM patients receiving temozolomide chemotherapy in addition to irradiation [2]. Several other molecular markers with prognostic and predictive significance in GBMs have been identified [24]. Except for a few recent reports on the role of miRNAs in GBM prognosis, the possibility of prognostic miRNA signatures have not been investigated [25]. To our knowledge, this is the first report of a miRNA expression signature predicting GBM patient survival.

The ten miRNA signature identified in this study included three miRNAs (miR-20a, miR-106a and miR-17-5p) that were protective and seven miRNAs (hsa-miR-31, hsa-miR-222, hsa-miR-148a, hsa-miR-221, hsa-miR-146b, hsa-miR-200b and hsa-miR-193a) that were risky with respect to their association between their expression and patient survival. The protective miRNAs were expressed at a higher level in the low risk group compared to the high risk group and the risky miRNAs were expressed at a higher level in the high risk group than in the low risk group. The protective and risky nature of these miRNAs is suggestive of their functions being either inhibitory or promoting, respectively, of various properties of cancer cells like proliferation, migration and invasion etc.

miR-31 has been shown to be an inhibitor of breast cancer metastasis by targeting RhoA, RDX and ITGA which are involved in tumor motility, invasion and resistance to anoikis [26]. However, miR-31 has also been shown to be an oncogenic miRNA in lung cancer by targeting tumor suppressor genes and in head and neck cancer by targeting factor-inhibiting hypoxia-inducible factor (FIH) [27], [28]. Both miR-221 and 222 have shown to be upregulated in multiple cancers including glioblastoma, linked to promoting proliferation and radioresistance by targeting PTEN, p27 and p57 [29]–[31]. Overexpression of miR-221 and miR222 has been shown to be associated with poor survival in hepatocellular carcinoma, pancreatic cancer and cervical cancer [32]–[34]. Further, a low expression of p27Kip1, a target of miR-221 and 222, has been correlated to poor prognosis in astrocytoms [35]–[37]. miR-148a has been shown to regulate DNA methylation by targeting DNA methylatransferase 1 (DNMT1) [38]. While Chou et al. [39] reported higher expression of miR-146b in high risk adult papillary thyroid carcinoma, other reports indicate miR-146b is a metastatsis suppressor by targeting matrix metalloproteases [39]–[41]. Similar to our results, higher miR-146b levels is correlated to poor prognosis in squamous cell lung cancer [42]. With respect to miR-200b, while Xia et al identified it to promote S-phase entry by targeting RND3 and increasing cyclin D1 expression, other reports suggest miR-200 to be an inhibitor of epithelial-to mesenchymal transition with decreased cell migration and increased sensitivity to EGFR-blocking agents [40], [43]. In malignant cutaneous melanoma, while increased expression of miR-193a is associated with poor survival, miR-193a has been identified as epigenetically silenced tumor suppressor miRNA in oral cancer [44], [45].

miR-106a, one of the protective miRNAs was found to be overexpressed and oncogenic in human T-Cell leukemia [46]. However in good correlation with our data, low expression of miR-106a was found to be associated with poor patient survival in glioma and colon cancer [25], [47]. The miR-17∼92 cluster, which contains two protective miRNAs, miR-17-5p and miR-20a, has been found to accelerate the disease onset of Eµ-myc-induced B-cell lymphoma, promote lung cancer growth *in vitro*, activated by c-myc and promote tumor angiogenesis [48]. Interestingly, in breast cancer, ovarian cancer and melanoma, miR-17∼92 has been shown to be deleted and found to inhibit cell proliferation upon overexpression suggesting miR-17∼92 cluster may have context dependent functions [48]. Our results show that both miR-17-5p and miR-20a are upregulated in GBMs with higher expression correlating with increased survival. In good correlation with our data, lower expression of E2F1, a target of miR-20a and cyclin D1, a target of both miR-20a and miR-17 was found to predict longer survival in gliomas [49]–[51].

Even though the median survival time remains in the 12–15 months range, the prognosis of individual patients is variable and approximately 10% of the patients are known to survive for more than 2 years [52]. At present, several molecular markers, including MGMT promoter methylation status for GBM patient prognosis, have been identified and many needs further validation before their use in clinical settings [24]. The ten miRNA signature, identified in this study, classifies patients successfully into low risk and high risk groups in both training and testing sets. This may help clinicians to identify patients belonging to high risk for more effective adjuvant therapy in addition to the standard treatment protocol. We also found that the ten miRNA signature is an independent predictor of GBM patient survival.

Our finding that ten miRNA signature can predict GBM patient's survival also likely to generate potential molecular targets for the development of anticancer therapy. Since miRNAs can target multiple genes, more thorough studies are needed to understand the mechanism of action of these miRNAs which is likely to result in better understanding of glioma. In conclusion, we have found a ten miRNA signature that can predict GBM patient survival with a lot of potential prognostic and therapeutic implications for the GBM patient management.

## Materials and Methods

### TCGA miRNA dataset and patient information

miRNA expression data and the corresponding clinical data for glioblastoma samples were downloaded from The Cancer Genome Atlas (TCGA) data portal. The level 1 raw data for 364 samples, which included 354 GBM and 10 normal samples, on the Agilent Human 8x15K miRNA platform was downloaded from the TCGA data portal in July 2010. The raw data was quantile normalized and log2 transformed, and the probe-centric signals were converted to gene-centric signals using the AgiMicroRNA package in R software [53]. After filtering out genes that were not detected, there were a total of 305 miRNAs for the 364 samples. We then calculated the mean value for the normal samples for each miRNA, and subtracted this value from all the samples for that miRNA, to come up with a log2 ratio of GBM vs normal.

We obtained clinical information for those patients who had survival information. We only selected those patients who had undergone both radiotherapy and some form of chemotherapy. In addition, we eliminated patients who had Karnofsky's score lesser than 70, and survival time lesser than 30 days, since these patients might have died for reasons other than the disease itself. A total of 222 patients who fit these criteria were included for further analysis.

### Statistical analysis

The 222 samples were randomly assigned to a training data set (n = 111) or a testing data set (n = 111). The expression level of each miRNA (n = 306) was assessed by Cox regression analysis using the BRB array tools [54] package in the training set. Parametric test (p≤0.01) identified a set of miRNAs significantly correlated with survival. Using the permutation test method, with 10,000 permutations, we found 10 miRNAs were strongly correlated with survival (p≤0.005).

Using these 10 significant miRNAs, we constructed a formula that would predict survival. This formula was devised using the Cox regression coefficients derived from the Cox proportional hazard analysis. Specifically, we assigned each patient a risk score that is a linear combination of the expression levels of the significant miRNAs weighted by their respective Cox regression coefficients [55]. According to this model, patients having high risk scores are expected to have poor survival outcomes as compared to patients having low risk scores. The risk scores are calculated as follows:

Risk score  = (−0.39 x expression of hsa-mir-20a) + (−0.41 x expression of hsa-mir-106a) + (−0.39 x expression of hsa-mir-17-5p) + (0.28 x expression of hsa-mir-31) + (0.23 x expression of hsa-mir-222) + (0.19 x expression of hsa-mir-148a) + (0.24 x expression of hsa-mir-221) + (0.22 x expression of hsa-mir-146b) + (0.19 x expression of hsa-mir-200b) + (0.29 x expression of hsa-mir-193a).

The significant miRNAs that formed the signature were of two types - risky and protective. Risky miRNAs were defined as those that had hazard ratio for death greater than 1. Protective miRNAs were defined as those that had hazard ratio for death less than 1. Using this definition, we found 3 protective miRNAs and 7 risky miRNAs.

We divided patients in the training data set into high-risk and low-risk groups by risk score. We used the 60^th^ percentile risk score as the cut-off, since this divided the training set patients into two groups having different survival times with highest significance. The Kaplan-Meier method was used to estimate overall survival time for the two groups. Differences in survival times were analyzed using the two-sided log rank test. We followed the strategy of splitting patients into training and testing sets, as we had no independent cohort that we could verify our signature with. We used the splitting strategy as opposed to cross-validation, since this has been found to be a better strategy [56]. We also used Cox multivariate analysis to evaluate the contribution of patient age as an independent prognostic factor. The miRNA risk score and age were used in the multivariate analysis.

## Supporting Information

Figure S1 study period in the training, the testing and the entire patient sets.
(TIF)
