# Supplementary figures and images for: A Ten-microRNA Expression Signature Predicts Survival in Glioblastoma
Source: PLoS One. 2011 Mar 31;6(3):e17438. doi: 10.1371/journal.pone.0017438 (PMC3069027; doi:10.1371/journal.pone.0017438)

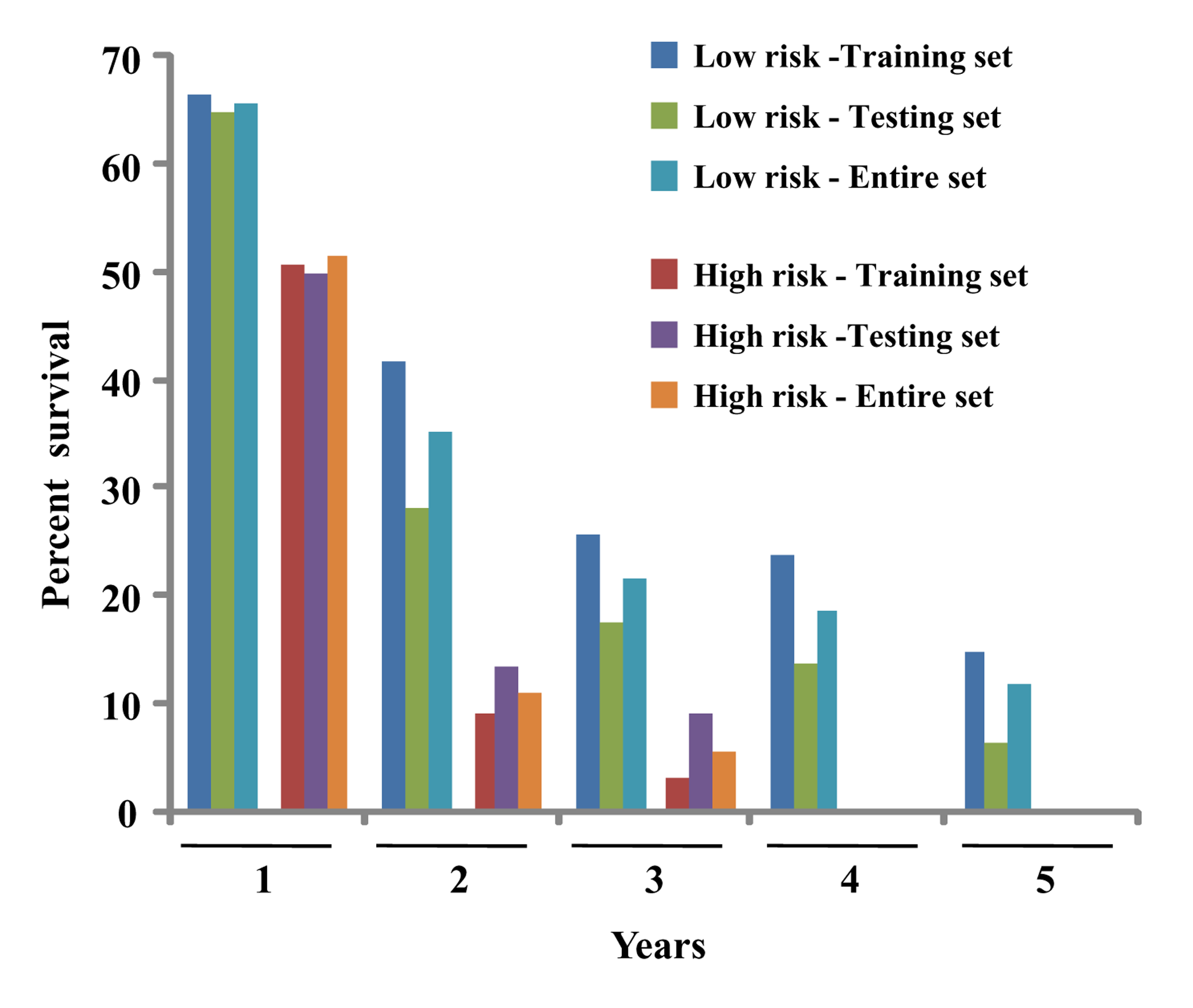

Supplement: Figure S1 — study period in the training, the testing and the entire patient sets. (TIF) [file pone.0017438.s001.tif]
